# SGLT2 Inhibitors Modify Fibrosis-4 Index and Mitigate the Development of DKD: Role of Background Antidiabetic Drugs

**DOI:** 10.1210/jendso/bvaf135

**Published:** 2025-08-19

**Authors:** Keisuke Takano, Koichi Hayashi, Koichi Kitamura, Mutsuo Kanda, Kei Ito, Taro Hirai, Yuki Hara, Akihiro Miyake, Keita Endo, Kaede Yoshino, Shinsuke Ito, Shigeki Fujitani, Toshihiko Suzuki

**Affiliations:** Department of Nephrology, Endocrinology and Diabetes, Tokyo Bay Urayasu Ichikawa Medical Center, Urayasu, 3-4-32 Todaijima Urayasu-city, Chiba 2790001, Japan; Department of Emergency and Critical Care Medicine, St Marianna University School of Medicine, 2-16-1 Sugao Miyamae-ku Kawasaki-city, Kanagawa 2168511, Japan; Department of Nephrology, Endocrinology and Diabetes, Tokyo Bay Urayasu Ichikawa Medical Center, Urayasu, 3-4-32 Todaijima Urayasu-city, Chiba 2790001, Japan; Department of Nephrology, Endocrinology and Diabetes, Tokyo Bay Urayasu Ichikawa Medical Center, Urayasu, 3-4-32 Todaijima Urayasu-city, Chiba 2790001, Japan; Department of Nephrology, Endocrinology and Diabetes, Tokyo Bay Urayasu Ichikawa Medical Center, Urayasu, 3-4-32 Todaijima Urayasu-city, Chiba 2790001, Japan; Department of Nephrology, Endocrinology and Diabetes, Tokyo Bay Urayasu Ichikawa Medical Center, Urayasu, 3-4-32 Todaijima Urayasu-city, Chiba 2790001, Japan; Department of Nephrology, Endocrinology and Diabetes, Tokyo Bay Urayasu Ichikawa Medical Center, Urayasu, 3-4-32 Todaijima Urayasu-city, Chiba 2790001, Japan; Department of Nephrology, Endocrinology and Diabetes, Tokyo Bay Urayasu Ichikawa Medical Center, Urayasu, 3-4-32 Todaijima Urayasu-city, Chiba 2790001, Japan; Department of Nephrology, Endocrinology and Diabetes, Tokyo Bay Urayasu Ichikawa Medical Center, Urayasu, 3-4-32 Todaijima Urayasu-city, Chiba 2790001, Japan; Department of Nephrology, Endocrinology and Diabetes, Tokyo Bay Urayasu Ichikawa Medical Center, Urayasu, 3-4-32 Todaijima Urayasu-city, Chiba 2790001, Japan; Department of Nephrology, Endocrinology and Diabetes, Tokyo Bay Urayasu Ichikawa Medical Center, Urayasu, 3-4-32 Todaijima Urayasu-city, Chiba 2790001, Japan; Department of Emergency and Critical Care Medicine, St Marianna University School of Medicine, 2-16-1 Sugao Miyamae-ku Kawasaki-city, Kanagawa 2168511, Japan; Department of Nephrology, Endocrinology and Diabetes, Tokyo Bay Urayasu Ichikawa Medical Center, Urayasu, 3-4-32 Todaijima Urayasu-city, Chiba 2790001, Japan

**Keywords:** fibrosis-4 index, SGLT2 inhibitors, metformin, diabetic kidney disease, type 2 diabetes mellitus

## Abstract

**Context:**

The fibrosis-4 (FIB-4) index is a noninvasive marker for liver fibrosis and is associated with the occurrence of diabetic kidney disease (DKD). Although sodium-glucose cotransporter-2 inhibitors (SGLT2is) are recognized to provide cardiovascular and renal benefits, the role of SGLT2is in regulating FIB-4 index and the subsequent effect on renal events remain unclear.

**Objective:**

This work aimed to clarify whether the FIB-4 index was associated with the development of DKD (estimated glomerular filtration rate < 60 mL/min/1.73 m^2^ or new-onset macroalbuminuria) and whether the background treatment with other antidiabetic drugs modified the effects of SGLT2i on FIB-4 index and renal events.

**Methods:**

A retrospective cohort study was conducted that included 136 patients with type 2 diabetes mellitus who were newly given SGLT2is for 3 years.

**Results:**

Patients with a high FIB-4 index (≥1.3) at baseline exhibited a higher incidence of de novo DKD, compared to those with a lower FIB-4 index (<1.3). This association was ameliorated in patients whose FIB-4 index was decreased during the SGLT2i treatment. Finally, among the background therapies, metformin use was associated with a decrease in FIB-4 index and a lower cumulative incidence of de novo DKD.

**Conclusion:**

Elevated FIB-4 index at baseline is a potent predictor for developing DKD, and combination therapy with SGLT2is and metformin may enhance renal protection by modulating FIB-4 index.

Metabolic dysfunction–associated steatotic liver disease (MASLD), formerly known as a nonalcoholic fatty liver disease, is the most common form of liver disease [1, 2] and is associated with morbidity and mortality as liver fibrosis develops [3, 4]. Substantial evidence has accumulated that the fibrosis-4 (FIB-4) index, which can be calculated using routinely available parameters, including age, platelet count, aspartate transaminase and alanine transaminase, is a noninvasive and potentially alternative tool that evaluates the possibility of developing liver fibrosis [5, 6]. Recently, the correlation between changes in FIB-4 index and renal function during a 3-year observational period has been demonstrated in MASLD patients [7] and a growing number of studies aim to clarify whether the FIB-4 index predicts the development of chronic kidney disease (CKD) [7, 8] and diabetic kidney disease (DKD) [9-11]. The putative mechanism why the liver fibrosis marker predicts the development of kidney disease is that common risk factors, including hypertension, insulin resistance, dyslipidemia, and obesity, are highly prevalent among patients with steatotic liver disease and those with CKD [12]. Although these observations suggest the FIB-4 index as a prognostic and/or surrogate marker for CKD and DKD, it remains undetermined what factors or preceding therapies affect the FIB-4 index and renal function in patients with type 2 diabetes mellitus (DM).

To date, a number of antidiabetic drugs with diverse mechanisms of glycemic control have been developed. Of these, the sodium-glucose cotransporter-2 inhibitor (SGLT2i) exerts euglycemic action by inhibiting the reabsorption of glucose at the proximal tubule and is often administered as an add-on therapy to other antidiabetic drugs. The additive benefits of SGLT2is on the cardiovascular system are well established, and many studies have elucidated the renal protective action of SGLT2is in patients with both DKD [13-15] and non-DM CKD [16]. Moreover, SGLT2is can lower the FIB-4 index [17, 18] and may alleviate the progression of DKD. In contrast, SGLT2is are also reported to exert variable effects on the FIB-4 index in patients with type 2 DM [19], thus suggesting apparently inconsistent effects of SGLT2is on FIB-4 index. Hence, the mechanisms underlying the action of SGLT2is on the FIB-4 index are not well understood. Furthermore, little is known concerning the ability of the FIB-4 index to predict the renal beneficial action of SGLT2is or combination therapy with other antidiabetic drugs.

In this study, we evaluated the effects of 3-year SGLT2i treatment on changes in FIB-4 index and renal function in patients with type 2 DM. The aim of this study was to clarify whether FIB-4 index and their changes during the observation period played a role in detecting the development of DKD. Furthermore, whether the background treatment with other antidiabetic drugs modified the effect of SGLT2is on FIB-4 index and the incidence of DKD was also assessed.

## Materials and Methods

This study is a retrospective cohort analysis evaluating the effect of SGLT2is on FIB-4 index and renal function in patients with type 2 DM during a 3-year observational period. The primary outcome was whether a high FIB-4 index and their changes were associated with the development of DKD, defined as either a decrease in estimated glomerular filtration rate (eGFR) to less than 60 mL/min/1.73 m^2^ or a new-onset macroalbuminuria (urinary albumin/creatinine [U-Alb/Cr] ≥300 mg/g·Cr), during treatment with SGLT2is.

The study was approved by the ethics committee of Tokyo Bay Urayasu Ichikawa Medical Center with a waiver of the requirement for obtaining informed consent (approval No. 967) and was registered at UMIN (ID; UMIN000037043). The opt-out information was published on our hospital website. The study was conducted in accordance with the Declaration of Helsinki. Information from medical records was anonymized prior to final analysis.

### Patient Enrollment

We enrolled 251 patients with type 2 DM (age ≥18 years) who were newly given SGLT2is at the outpatient clinic of Tokyo Bay Urayasu Ichikawa Medical Center between April 2014 and September 2018. Eligible individuals were either treatment naive (ie, no glucose-lowering drugs) or on antidiabetic therapy before administration of SGLT2is with a glycated hemoglobin A1c (HbA1c) between 7.0% and 11.0%. Patients on steroid therapy or immunosuppressive agents were excluded from the study. We also excluded individuals who could not be followed for more than 3 years (mainly due to referral to other clinics) and individuals with multiple missing data. Finally, the patients with reduced renal function (eGFR <60 mL/min/1.73 m^2^) were excluded. Ultimately, 136 patients were enrolled in this study.

### Laboratory Analysis

The effects of SGLT2is on FIB-4 index and other biochemical parameters, including HbA1c, serum creatinine, and U-Alb/Cr, were assessed over 36 months. Anthropometric parameters (blood pressure [BP] and body mass index [BMI]) as well as eGFR were also evaluated following the administration of SGLT2is. eGFR was calculated using the formula adapted to the Japanese population [20]:


$$\begin{array}{rl} \mathrm{eGFR}\;= & 194\;\times \;\mathrm{ag}e^{-0.287}\\ & \times \;\mathrm{serum}\;\mathrm{creatinin}e^{-1.094}(\times 0.739,\mathrm{if}\;\mathrm{female}) \end{array}$$

The FIB-4 index was calculated as follows:


$$\begin{array}{rl} \mathrm{FIB}\text{-}4\;\mathrm{index}\;= & \;\mathrm{age}\;(\mathrm{years})\times \mathrm{AST}\;(U/L)/[\mathrm{ALT}\;(U/L{)}^{1/2}\\ & \times \mathrm{platelet}\;\mathrm{count}\;(10^{9}/L)] \end{array}$$

FIB-4 index was categorized as low risk (FIB-4 index <1.3; low FIB-4 index) or high risk (FIB-4 index ≥1.3; high FIB-4 index) of advanced liver fibrosis [5, 6].

Serial changes in eGFR and cumulative incidence of DKD (eGFR <60 mL/min/1.73 m^2^ or U-Alb/Cr ≥300 mg/g·Cr) were assessed based on FIB-4 index at baseline and the trend in FIB-4 index changes from baseline (ie, increases or decreases in FIB-4 index during 3 years). Finally, the effects of the background therapy with other antidiabetic drugs (ie, metformin, GLP-1 receptor agonists [GLP-1 RAs]), pioglitazone, insulin, sulfonylurea, and DPP4 inhibitors [DPP4is]) on serial changes in eGFR and the cumulative incidence of DKD were assessed during the 3-year treatment with SGLT2is.

### Statistical Analysis

The results are expressed as the median (lower quartile-upper quartile). Data were compared with the Mann-Whitney *U* test or Kruskal-Wallis test, as appropriate. The chi-square or Fisher exact test was used to compare categorical variables, including the number of patients. Odds ratios (ORs) for the number of patients with the decrease in FIB-4 index at 3 years of SGLT2i administration were calculated in association with various anthropometric parameters and antidiabetic drugs, using multivariate logistic regression analysis. To reduce the confounding effects of the covariables affecting the outcomes following categorization based on the baseline FIB-4 index, the source data were reanalyzed after propensity score matching. A logistic regression model was applied to generate propensity scores for categorized groups by baseline FIB-4 index, with demographic and eGFR as independent variables, and caliper width was set at 0.2 or less of the SD of the logit of the propensity score. The matched pairs were then analyzed with the Wilcoxon signed-rank test.

Kaplan-Meier analyses and log-rank tests were used to compare the cumulative hazard of renal dysfunction (ie, eGFR <60 mL/min/1.73 m^2^ or macroalbuminuria) during the follow-up between individuals with low and high FIB-4 index. Statistical analyses were performed using the John Macintosh Project (JMP) statistical software (version 17, SAS Institute Inc). Statistical significance was set at *P* less than .05.

## Results

### Baseline Characteristics

Among 136 patients enrolled in this study, 48 (35.3%) and 88 patients (64.7%) were categorized as a high FIB-4 index and a low FIB-4 index group, respectively. The SGLT2is used in this study were tofogliflozin (n = 2), canagliflozin (n = 18), empagliflozin (n = 12), ipragliflozin (n = 40), dapagliflozin (n = 54), and luseogliflozin (n = 10).

Patients with a high FIB-4 index were older than those with a low FIB-4 index (*P* < .001; Table 1). There were no statistically significant differences in BMI, systolic BP (SBP), diastolic BP, eGFR, or U-Alb/Cr between these groups. Metformin was prescribed as a background antidiabetic drug in 93 patients (68.3%) and was given more frequently among the patients with a low FIB-4 index (79.6% vs 47.9%, for low FIB-4 index and high FIB-4 index, respectively, OR = 4.23 [95% CI, 1.96-9.11]; *P* < .001).

**Table 1.** Baseline characteristics of patients

	FIB-4 index		
	High (≥1.3, n = 48)	Low (<1.3, n = 88)	*P*
Age, y	65.5 (54.0-71.0)	50.5 (45.3-59.0]	<.001
Male, n (%)	31 (64.6%)	56 (63.6%)	
BMI	26.3 (23.1-31.0)	27.6 (24.8-32.0)	
Systolic BP, mm Hg	137 (127-148)	133 (125-146)	
Diastolic BP, mm Hg	79 (66-87)	80 (71-88)	
AST, U/L	27 (22-41)	19 (15-24)	<.001
ALT, U/L	25 (18-43)	26 (16-37)	
Platelets, × 10^4^/µL	20.1 (17.8-23.7)	24.9 (22.2-27.7)	<.001
LDL cholesterol, mg/dL	104 (79-127)	110 (93-126)	
Serum albumin, g/dL	4.0 (3.7-4.2)	4.2 (4.1-4.4)	<.001
Estimated GFR, mL/min/1.73 m^2^	77 (69-88)	84 (70-95)	
U-Alb/Cr, mg/g·Cr	65 (19-202)	26 (12-118)	
HbA1c, %	8.3 (7.5-9.7)	7.9 (7.2-9.0)	
FIB-4 index	1.64 (1.49-1.95)	0.84 (0.62-1.07)	<.001
Cerebrovascular disease, n (%)	2 (4.2%)	5 (5.7%)	
Cardiovascular disease, n (%)	14 (29.2%)	15 (17.1%)	
Drugs			
Metformin, n (%)	23 (47.9%)	70 (79.6%)	<.001
Pioglitazone, n (%)	3 (6.3%)	4 (4.5%)	
GLP-1 RA, n (%)	6 (12.5%)	8 (9.1%)	
Insulin, n (%)	23 (47.9%)	32 (36.4%)	
Sulfonylurea, n (%)	3 (6.3%)	9 (10.2%)	
DPP4i, n (%)	36 (75.0%)	65 (73.9%)	
RAS inhibitors, n (%)	25 (52.1%)	43 (48.9%)	

Data in brackets denote interquartile ranges.

Abbreviations: ALT, alanine transaminase; AST, aspartate transaminase; BMI, body mass index; BP, blood pressure; DPP4i, DPP-4 inhibitor; FIB-4, fibrosis-4; GFR, glomerular filtration rate; GLP-1 RA, GLP-1 receptor analogue; HbA1c, glycated hemoglobin A1c; LDL, low-density lipoprotein; U-Alb/Cr, urinary albumin to creatinine ratio; RAS, renin-angiotensin system.

### Temporal Changes in Biophysical/Biochemical Parameters During Sodium-Glucose Cotransporter-2 Inhibitor Treatment

The treatment with SGLT2is reduced HbA1c both in the high FIB-4 index (from 8.3 [interquartile range (IQR): 7.5-9.7] to 7.5 [IQR: 7.0-8.3]% at 12 months; *P* < .001) and low FIB-4 index groups (from 7.9 [IQR: 7.2-9.0] to 7.2 [IQR: 6.6-8.0]% at 12 months; *P* < .001), and the effects were sustained throughout the 3-year observation period. However, no temporal changes in FIB-4 index were observed in either the high FIB-4 index or the low FIB-4 index group. Similarly, there were no statistically significant differences in SBP, diastolic BP, or U-Alb/Cr between the high and the low FIB-4 index groups during the observation period.

**Figure 1.**
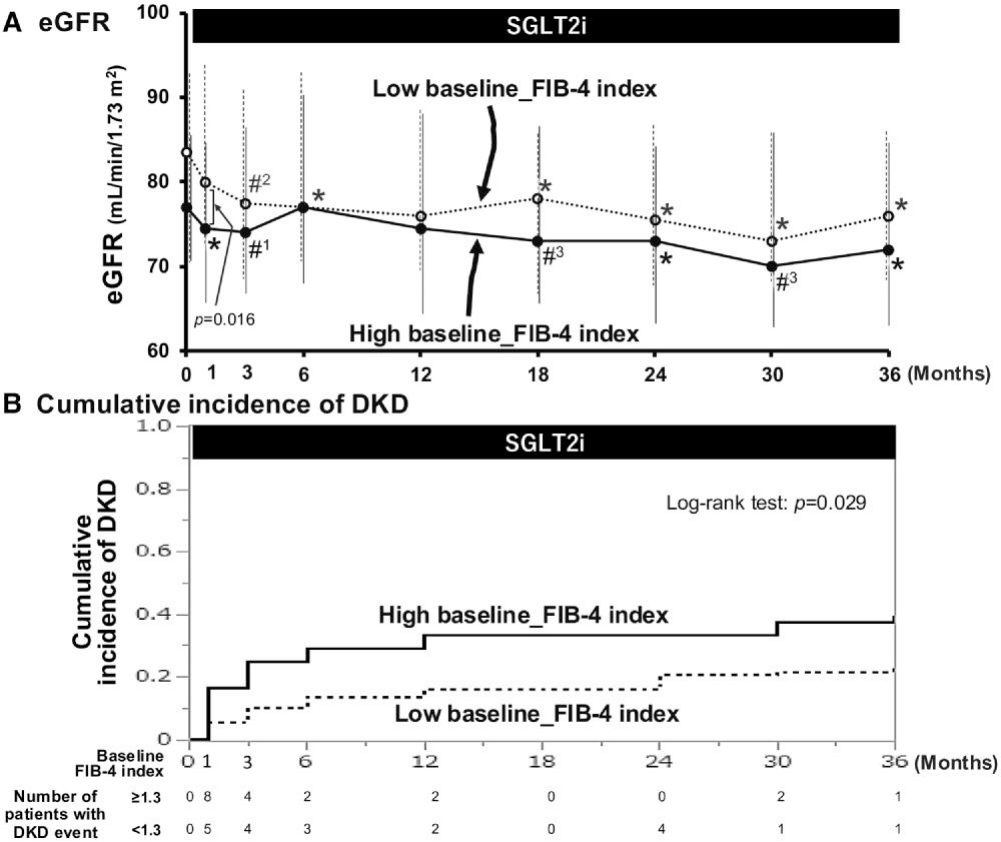
Temporal changes in kidney function during SGLT2i treatment. (A) eGFR changes after SGLT2i initiation in patients with high and low FIB-4 index. A slight initial dip was seen, followed by stable levels of eGFR, with modest differences between these groups. *P < .001; #^1^*P* = .001; #^2^*P* = .002; #^3^*P* = .025 vs baseline (0 month). (B) A graph depicting the cumulative incidence of de novo DKD. Patients with a high baseline FIB-4 index showed a higher risk than those with a low index (log-rank test: *P* = .029).

In both the high and the low FIB-4 index groups, the administration of SGLT2is resulted in a slight fall in eGFR at 1 month, followed by nearly constant eGFR thereafter (Fig. 1A); there was a modest difference in eGFR between the high and the low FIB-4 index group at each time point, and the yearly changes in eGFR were nearly the same between these groups (−1.5 vs −1.4 mL/min/1.73 m^2^/year, respectively; *P* = .872). The cumulative incidence of the development of DKD (decline in eGFR to <60 mL/min/1.73 m^2^ or de novo macroalbuminuria), however, was higher among patients with a high FIB-4 index (log-rank test: *P* = .029; Fig. 1B).

Because there was a marked difference in age between the high and the low FIB-4 index groups at baseline (*P* < .001; see Table 1), the cumulative incidence of DKD was reevaluated after propensity score matching, with age, BMI, SBP, and eGFR as independent variables. Thus, after adjustment of age between the two groups (62 [IQR: 53-68] vs 61 [IQR: 50-67] years, n = 35, for high and low FIB-4 index groups, respectively), Kaplan-Meier analysis showed no significant difference in the cumulative incidence rate of renal events (log-rank test: *P* = .310).

### Trends in Changes of Fibrosis-4 Index and Association With Background Antidiabetic Drugs

**Figure 2.**
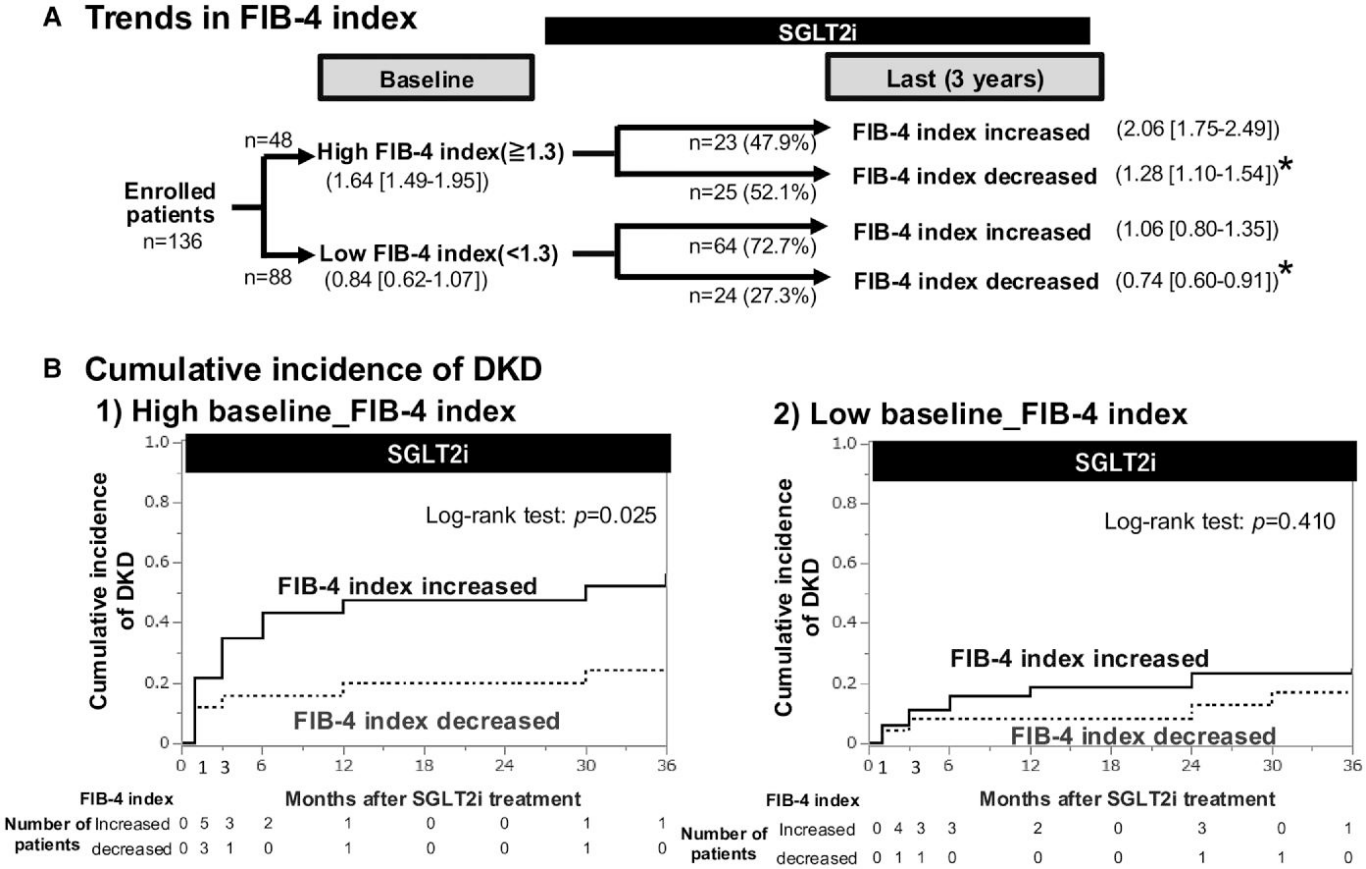
Effects of trends in FIB-4 during SGLT2i treatment and cumulative incidence of DKD. Among the subgroup with a high baseline FIB-4 index (*n* = 48), 25 patients (ie, 52.1%) showed a decrease in FIB-4 after 3-year treatment with SGLT2i (A). Kaplan–Meier analysis revealed that this subgroup of patients had a lower risk of developing DKD than that with an increased FIB-4 index (log-rank test: *P* = .025, B). Data in brackets denote interquartile ranges. *P < .001 vs FIB-4 increased.

Of the patients with a high FIB-4 index at baseline (n = 48), 25 patients (52.1%) manifested a decrease in FIB-4 index at 3 years of SGLT2i treatment (Fig. 2A). In this subgroup, the cumulative incidence of de novo DKD was lower than in patients with increased FIB-4 index (log-rank test: *P* = .025; Fig. 2B); at baseline, neither age (65 [IQR: 54-69] vs 68 [IQR:53-73] years) nor eGFR (74 [IQR: 64-87] vs 77 [IQR: 73-91] mL/min/1.73 m^2^) differed between these subgroups. Among the patients with a low baseline FIB-4 index (n = 88), 64 patients (72.7%) showed an increase in FIB-4 index at 3 years of SGLT2i treatment. However, no difference in the cumulative incidence was noted between the subgroups with different trends in FIB-4 index (log-rank test: *P* = .410).

We then examined the patients with a high baseline FIB-4 index to elucidate what factors influenced the trend in FIB-4 index during the study period. Neither age (age < 65 vs ≥65 years; OR = 1.88 [95% CI, 0.45-7.87]), sex (male vs female; OR = 1.68 [95% CI, 0.47-5.92]), nor BMI (<30 vs ≥30; OR = 1.58 [95% CI, 0.35-7.13]) affected the trends in FIB-4 index. Among the background antidiabetic drugs that had been used at the initiation of the study, metformin was the only drug most closely associated with the decrease in FIB-4 index (OR = 3.33 [95% CI, 0.89-12.45]; *P* = .074).

### Effect of Background Antidiabetic Drugs on Sodium-Glucose Cotransporter-2 Inhibitor–mediated Renal Protection

**Figure 3.**
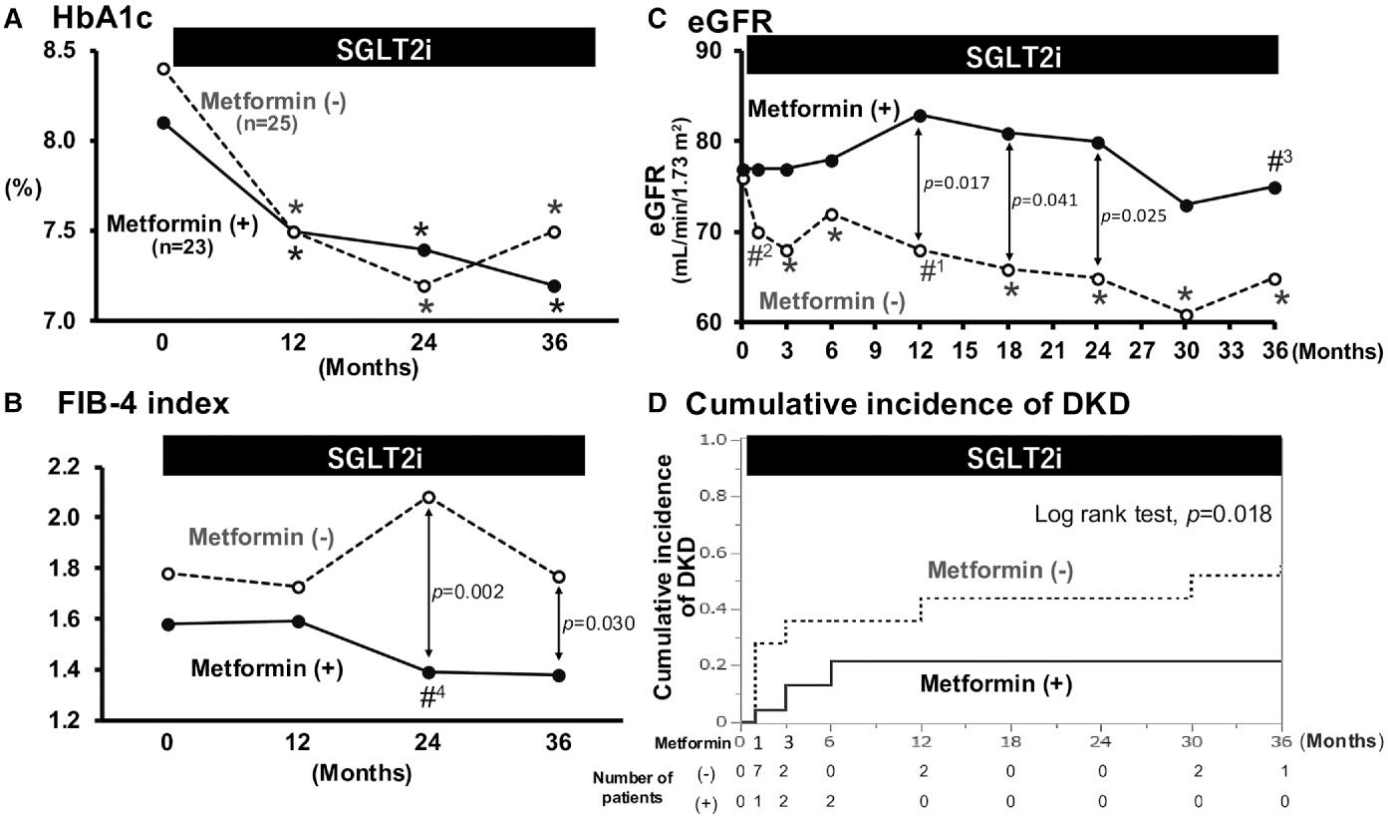
Temporal changes in various parameters during the SGLT2i treatment period in patients with high baseline FIB-4. Whereas SGLT2i caused similar decreases in HbA1c, FIB-4 was reduced in the metformin-treated group (A, B). Patients with metformin therapy did not manifest an initial dip, a response usually seen after SGLT2i treatment, but maintained a relatively stable time course of eGFR thereafter (C). Kaplan–Meier analysis showed that metformin users had a lower risk of developing DKD (log-rank test: *P* = .018). *P < .001, #^1^  *P* = .007, #^2^  *P* = .008, #^3^  *P* = .020, #^4^  *P* = .035 vs baseline (0 month).

Next, we examined whether underlying antidiabetic drugs modified the effect of SGLT2is on renal function in patients with a high baseline FIB-4 index. At baseline (0 M), there were no differences in age (62 [IQR: 52-69] vs 67 [IQR: 60-73] years; *P* = .137), FIB-4 index (1.58 [IQR: 1.40-1.78] vs 1.78 [IQR: 1.54-2.13]; *P* = .073), or eGFR (77 [IQR: 72-93] vs 76 [IQR: 64-85] mL/min/1.73 m^2^; *P* = .157) between the metformin-treated and metformin-untreated groups. While SGLT2is caused similar decreases in HbA1c in metformin-treated and metformin-untreated groups, FIB-4 index was reduced only in the metformin-treated group (Fig. 3A and 3B). In patients with no metformin therapy, SGLT2is elicited an initial dip in eGFR (from 76 [IQR: 64-85] to 70 [IQR: 57-84] mL/min/1.73 m^2^ at 0 and 1 month, respectively; *P* = .008) and offered a modest downslope trend in eGFR thereafter (ie, −2.6 mL/min/1.73 m^2^/year; Fig. 3C). In patients with underlying treatment with metformin, however, an initial dip was not observed and a relatively stable time course of eGFR was seen, with a yearly change in eGFR of −0.7 mL/min/1.73 m^2^/year (*P* = .165 vs metformin [–]). The cumulative incidence of DKD was lower in the group receiving metformin (log-rank test: *P* = .018; Fig. 3D).

In patients with a high baseline FIB-4 index, neither GLP-1 RA, insulin, nor DPP4is affected the temporal changes in eGFR during the 3 years of SGLT2i treatment (Supplementary Fig. S1 [21]). Likewise, the cumulative incidence of DKD did not differ between the treated and untreated subgroups of the respective drugs (see Supplementary Fig. S1 [21]).

## Discussion

Recent advances in the treatment of DM offer a new generation of antidiabetic drugs, among which SGLT2is are among the most crucial drugs prescribed frequently. Several studies have demonstrated that SGLT2is reduce FIB-4 index in patients with DM [17, 18], whereas other investigators showed no consistent changes in FIB-4 index with SGLT2is [19]. Furthermore, while the FIB-4 index contributes to the prediction of MASLD and liver fibrosis as a noninvasive marker, whether this parameter plays a similar role in the detection of renal damage in patients with DM remains to be determined [8-11, 19].

The present study demonstrated that a high FIB-4 index (≥1.3) at baseline was associated with the development of de novo DKD, including a decline in eGFR to less than 60 mL/min/1.73 m^2^ and a new onset of macroalbuminuria, during the 3 years of SGLT2i treatment (log-rank test: *P* = .029; see Fig. 1B). Although the mechanism underlying the association between FIB-4 index and renal impairment remains to be elucidated, several recent studies have shown that high FIB-4 index is associated not only with the development of CKD following a 5-year follow-up period in metabolically healthy men [8] but also with the incidence of CKD over 10 years in the general population [7]. Furthermore, Saito et al [11] demonstrated that the risk of developing DKD was higher in patients with a FIB-4 index greater than 1.3 during a 6 (median)-year observational period. Of note, our results on the cumulative incidence of DKD turned out to show no significant difference between the high and the low baseline FIB-4 index group when adjusted for age, BMI, SBP, and eGFR at baseline, using propensity score matching (log-rank test: *P* = .310). Hence, these observations and the previous findings indicate the need for more deliberate assessment.

There are divergent reports showing the long-term effect of SGLT2is on FIB-4 index. Arai et al [17] demonstrated that 48-week treatment with SGLT2is reduced FIB-4 index in patients with type 2 DM and MASLD, particularly with high FIB-4 index. Furthermore, 6-month or 12-month treatment with SGLT2is decreased the number of patients with high FIB-4 index [18]. In contrast, Corbin et al [19] reported no consistent changes in FIB-4 index with SGLT2is. In the present study, we found that after 3 years of treatment with SGLT2is, a decrease in FIB-4 index was more frequently seen in patients with a high FIB-4 index compared to the low FIB-4 index group at baseline (52.1% vs 27.3%; *P* = .004; see Fig. 2A). Thus, the ability of SGLT2is to decrease FIB-4 index may depend on the FIB-4 index prior to the administration of SGLT2is.

Whether the changes in FIB-4 index during long-term SGLT2is treatment affect the development of DKD remains unclarified. In the present study, we found that among patients with a high baseline FIB-4 index, the incidence of DKD was lower in the subpopulation who manifested a decrease in FIB-4 index after 3-year treatment with SGLT2is (see Fig. 2B). The decrease in FIB-4 index during the treatment period was not associated with age, sex, or BMI, but metformin was considered the most likely predictor of the decrease in FIB-4 index among the background antidiabetic drugs. These findings lend support to the premise that both baseline FIB-4 index and the tactics for DM management modify the course of kidney function even when appropriate glycemic control is achieved. Notably, 75.3% (70/93) of the patients receiving metformin showed a low FIB-4 index at baseline (see Table 1), thus suggesting a close link between metformin and FIB-4 index.

To confirm whether metformin and its interaction with SGLT2is played a role in decreasing the FIB-4 index and preventing the development of DKD, we evaluated the effect of SGLT2is on FIB-4 index and renal function in patients with a high baseline FIB-4 index. Thus, SGLT2is lowered FIB-4 index and reduced the cumulative incidence of DKD in patients who had been treated with metformin (see Fig. 3B and 3D). These novel findings suggest that metformin enhances the renal protective action of SGLT2is, particularly among patients with a high risk of developing DKD. In this regard, Brønden et al [22] have recently demonstrated that the addition of SGLT2is mostly to metformin therapy is less likely to lead to the development of composite renal outcomes (eGFR reduction of >30%-50%, eGFR <15, or renal replacement therapy) than DPP4is, using a network meta-analysis. In contrast, Neuen et al [23] demonstrated that SGLT2is reduced the risk of worsening kidney function or end-stage kidney disease, irrespective of whether metformin was used at baseline. Although our present study evaluated the interaction between metformin and SGLT2is in patients with a high FIB-4 index at baseline, the cumulative incidence of DKD was approximately the same in patients receiving metformin and those not receiving it when assessed among the group with a low FIB-4 index (log rank test: *P* = .487) and the entire population (*P* = .052). It is surmised therefore that the role of metformin in the SGLT2i-induced effect on renal function differs depending on the risk level of the patients as well as the renal end point measures of the respective studies.

Of note, SGLT2is activate hypoxia-inducible factor (HIF)-2α and suppress inflammation and fibrosis [24], part of which may involve the inhibition of macrophage infiltration [24]. Likewise, metformin possesses anti-inflammatory action [25] and inhibits fibrotic changes in the liver [26]. Thus, the combined treatment with SGLT2is and metformin may enhance the inhibition of the inflammatory and fibrotic processes in the kidney and is actually reported to offer an additive beneficial action on renal histology [27], which may be associated with the preserved eGFR in patients with DM [26, 28]. Finally, many studies, including our present and previous studies as well as those of other investigators, have demonstrated that metformin attenuates the initial decline in eGFR normally observed after SGLT2i administration (see Fig. 3C) [15, 28]. To the extent that an exaggerated initial decline in eGFR following SGLT2i treatment is associated with renal events [29], metformin appears to be teleologically preferred as a background antidiabetic medication to circumvent acute renal adverse events.

### Limitations

Since the present study was conducted in a single medical center, the characteristics of the patients might present some bias. Indeed, the prescription rate of GLP-1 RA was relatively small (10.3%), which prevented the detailed evaluation of the effect of this drug as a background therapy. Additionally, we could not conclude that the modulation of FIB-4 index was attributed solely to one background drug or to the combination of some other antidiabetic drugs. There were various combinations of antidiabetic drugs, and the sample size was not large enough to assess them. Furthermore, the calculation of FIB-4 index includes age as a variable [5]. Thus, categorization based on FIB-4 index may create subgroups with different ages [8, 11], which should affect age-dependent parameters such as eGFR [20] and hence may modify the incidence of renal events. Although we employed propensity score matching to alleviate this dilemma, more deliberate strategies may be required to counter this issue.

### Conclusions

The present study demonstrated that despite adequate glycemic control by SGLT2is, highly sustained or elevated FIB-4 index was associated with the development of DKD. When added to the background treatment with metformin, SGLT2is may lower FIB-4 index and reduce the incidence of DKD. Our observations therefore support the premise that combination therapy with SGLT2is and metformin provides a beneficial effect on the prevention of DKD through a common mechanism shared in part by the FIB-4 index.

## Data Availability

Some or all data sets generated during and/or analyzed during the current study are not publicly available but are available from the corresponding author on reasonable request. Supplemental material to this article can be found online at https://doi.org/10.5281/zenodo.16014275.

## Clinical Trial Information

Clinical trial registration number UMIN000037043 (registered June 13, 2019).

## Abbreviations

BMI: body mass index
BP: blood pressure
CKD: chronic kidney disease
DKD: diabetic kidney disease
DM: diabetes mellitus
DPP4i: DPP-4 inhibitor
eGFR: estimated glomerular filtration rate
FIB-4: fibrosis-4
GFR: glomerular filtration rate
GLP-1 RA: GLP-1 receptor analogue
HbA1c: glycated hemoglobin A1c
IQR: interquartile range
MASLD: metabolic dysfunction–associated steatotic liver disease
OR: odds ratio
RAS: renin-angiotensin system
SBP: systolic blood pressure
SGLT2i: sodium-glucose cotransporter-2 inhibitor
U-Alb/Cr: urinary albumin to creatinine ratio
